# The association between ultra-processed food exposure and cognition in older adults

**DOI:** 10.1007/s11357-026-02269-w

**Published:** 2026-05-06

**Authors:** Sarah Gauci, Belayneh Mengist, Mojtaba Lotfaliany, Malcolm Forbes, Farzaneh Asoudeh, Deb Junyi Zhang, Suzanne G. Orchard, Alice J. Owen, Daniel Clayton-Chubb, Robyn L. Woods, John J. McNeil, Joanne Ryan, Michael Berk, Adrienne O’Neil, Mohammadreza Mohebbi

**Affiliations:** 1 Deakin Institute for Mental and Physical Health and Clinical Translation, Deakin University, Geelong, VIC Australia; 2 School of Public Health and Preventive Medicine, Monash University, Melbourne, VIC Australia; 3 College of Medicine and Health Sciences, Debre Markos University, Debre Markos, Ethiopia; 4 Department of Gastroenterology, Alfred Health, Melbourne, VIC Australia; 5 School of Translational Medicine, Monash University, Melbourne, VIC Australia; 6 Department of Gastroenterology, Eastern Health, Box Hill, VIC Australia; 7 Department of Gastroenterology, St Vincent’s Hospital Melbourne, Fitzroy, VIC Australia; 8 Biostatistics Unit, Faculty of Health, Deakin University, Geelong, VIC Australia

**Keywords:** Ultra-processed food, Dietary exposure, Lifestyle change, Cognition, Dementia, Neurosciences, Cognitive decline

## Abstract

With an ageing population comes a growing burden of age-related cognitive decline. While evidence supports a relationship between higher diet quality and better cognitive outcomes, the association between ultra-processed foods (UPFs) and cognitive function remains underexplored. We examined this association using prospective data from the ASPirin in Reducing Events in the Elderly (ASPREE) study, a cohort of Australian adults aged 70 years and older. Dietary intake was assessed via a mail-based diet screening questionnaire and categorised according to the Nova classification. Cognitive performance was assessed using the Controlled Oral Word Association Test (COWAT), Symbol Digit Modalities Test (SDMT), Hopkins Verbal Learning Test–Revised (HVLT-R), Modified Mini-Mental State Examination (3MS), and a composite cognitive *Z* score. Participants were stratified into high UPF (≥ 4 servings/day) and low UPF (< 4 servings/day). Marginal structural models with inverse probability of treatment weighting estimated associations, adjusting for demographic, lifestyle, and clinical confounders. Among 11,502 participants (3505 high UPF; 7997 low UPF), over a median follow-up of 5.6 (± 2.4) years, higher UPF consumption was associated with poorer performance on the 3MS (mean difference –0.28, 95% CI –0.52 to –0.04), COWAT (–0.23, 95% CI –0.44 to –0.03), SDMT (–0.51, 95% CI –0.94 to –0.08), and the composite *Z* score (–0.04, 95% CI –0.07 to –0.01), but not HVLT-R (–0.12, 95% CI –0.26 to 0.02). Higher UPF intake was associated with poorer cognitive performance across multiple domains. These findings support initiatives to reduce UPF exposure as a potential strategy to promote healthy cognitive ageing.

**Supplementary Information:**

The online version contains supplementary material available at https://doi.org/10.1007/s11357-026-02269-w.

## Introduction

The global population is rapidly ageing [1], leading to growing concern about cognitive ageing. Cognitive ageing refers to the gradual decline in cognitive abilities, such as memory, attention, processing speed, and executive function, and is associated with an increased risk of dementia [2]. While dementia represents a disabling endpoint of cognitive ageing, cognitive ageing also impacts quality of life [3]. Importantly, there are currently no effective treatments to stop or reverse cognitive ageing and halt dementia progression, making the maintenance of cognitive health across the lifespan a public health priority. Evidence suggests that up to 45% of dementia risk is attributable to modifiable risk factors [4–6]. Among these, diet plays an important role, influencing cognitive ageing both directly and through its impact on other risk factors, making it a promising target for the maintenance of cognitive function [7–10].

Considerable research has examined the role of diet in the pathogenesis and prevention of cognitive ageing. While findings remain varied, there is evidence that healthful dietary patterns, such as the Mediterranean diet, the DASH (Dietary Approaches to Stop Hypertension) diet, and the MIND (Mediterranean-DASH Intervention for Neurodegenerative Delay) diet, maintain cognitive function [11]. A recent large-scale randomised controlled trial examining a structured lifestyle intervention, including adherence to the MIND diet, resulted in improvements in cognitive function, compared with a lower-intensity self-guided approach [12]. These findings highlight the potential of healthful dietary patterns, particularly when combined with other lifestyle changes, in maintaining cognitive function. However, the impact of the modern Western diet, which is partly characterised by higher consumption of ultra-processed foods (UPFs), is less well understood [11].

Ultra-processed foods, as defined by the Nova classification system, are made up of industrial formulations containing little to no whole foods [13]. They include high quantities of sugars, fat, and salt, as well as additives like flavouring, colours, emulsifiers, and preservatives [13]. A recent umbrella review found that UPF consumption was related to adverse health outcomes, including cardiometabolic disease, common mental disorders, and mortality [14]. However, due to the lack of empirical evidence, outcomes for cognitive ageing were not included in the umbrella review.

The limited available research suggests a complex relationship between UPFs and cognitive ageing. For example, in a prospective cohort of older adults with type 2 diabetes (*N* = 568, mean follow-up 5.3 ± 1.5 years), overall exposure to UPFs was not associated with measures of cognitive decline [15]. However, when exploring specific UPFs, a higher exposure to ultra-processed meats and oils/spreads was associated with increased decline in domains of executive function and global cognition [15]. Furthermore, higher UPF exposure has also been associated with poorer cognitive function [16]. Specifically, people with diets high in UPF had a 28% faster rate of global cognitive decline and a 25% greater decline in executive function compared to individuals in the first quartile of UPF consumption [17].

Research linking exposure to UPFs with cognitive function is still emerging, with most existing studies conducted in North America and South America, which limits generalisability to other populations. To address this gap, the current study investigates the association between UPF exposure and cognitive function in a large cohort of Australian community-dwelling older adults.

## Methods

### Study population

This study is a secondary analysis of the ASPREE (ASPirin in Reducing Events in the Elderly) trial, a double-blind, randomised, placebo-controlled primary prevention study that evaluated the effects of daily 100-mg aspirin on dementia- and disability-free life in relatively healthy, community-dwelling older adults. The ASPREE trial enrolled 19,114 participants aged 70 years and older between 2010 and 2014. At trial enrolment, eligible participants required a score of at least 78/100 on the Modified Mini-Mental State Examination (3MS). Full details of the trial are available elsewhere [18]. Follow-up after the trial phase continued through the ASPREE-eXTension (ASPREE-XT) study, which involved annual measurements of cognitive function [19].

**Fig. 1:**
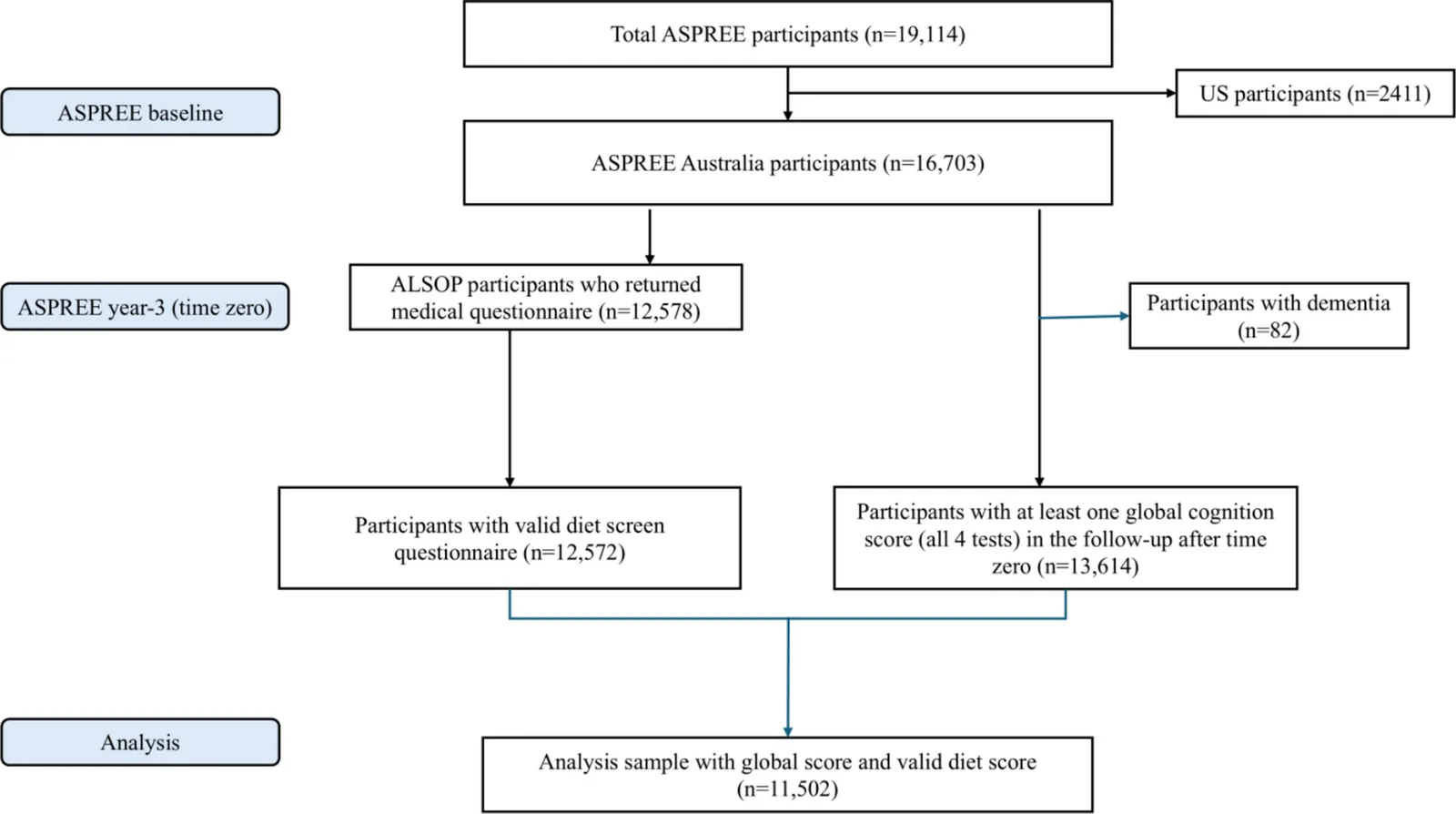
Study workflow diagram

For this analysis, dietary data were obtained from the ASPREE Longitudinal Study of Older Persons (ALSOP), a sub-study of ASPREE involving Australian participants (*n* = 14,892). At approximately annual visit 3 of ASPREE, 12,578 participants provided medical and lifestyle data, including a dietary screening survey administered as part of ALSOP. Accordingly, the ASPREE year 3 data collection visit was designated as the time zero for this analysis (see Fig. 1). Annual follow-up data collected through ASPREE and ASPREE-XT on cognitive function from years 4 to 11 were used as outcomes. Participants included were those who completed the food screening survey and had cognitive function measures in the annual follow-up visits. Participants with dementia at time zero were excluded. Further details of the ALSOP study are available elsewhere [20].

### Assessment of UPF consumption

Dietary data were captured using a mail-based diet screening questionnaire, which classified foods and beverages into categories such as meat, fish, and eggs; snacks and convenience foods; dairy (including milk and milk alternatives); bread, grains, and cereals; fruits and vegetables; drinks (e.g. soft drinks, cordial, supplement drinks); and other food items/nutrients (e.g. salt, fats and oils, water, discretionary foods). Participants were asked to report their frequency of consumption over the past 12 months, with responses ranging from “never” to “every day/several times a day”.

The level of food processing was assessed using the Nova food classification system [13, 21]. Items from the screening tool were divided into four groups: unprocessed/minimally processed (Nova group 1), culinary ingredients (Nova group 2), processed foods (Nova group 3), and UPFs (Nova group 4). Details on how the food and drink items were classified using the Nova classification system in this cohort have previously been published [22]. In brief, 21 food and drink items were classified as UPFs, the group of interest for this study (Nova group 4), including processed meats (e.g. bacon, ham, salami), sausages, potato chips, sweet biscuits, cakes, chocolates, lollies/sweets, fast food (e.g. hamburgers, pizza), meat pies, ice cream, breakfast cereals, crackers, mass-produced bread, soy or other non-dairy milk, malt drinks (e.g. Milo), cordial, soft drinks, diet sodas, and supplement drinks (e.g. Ensure) (Appendix Table 1).

Dietary data were then converted into a daily equivalent frequency for each food item, using a reference guide adapted from the Victorian Cancer Council, Australia, food frequency questionnaire. UPF consumption was calculated by converting frequency responses from the diet screen into daily servings. For foods, the scale ranged from 0 for “never” to 1 for “every day”. For drinks, the scale ranged from 0 for “never” to 3 for “three or more times a day”. The total daily servings of UPF for each participant were determined by summing the serving scores for each UPF item. UPF consumption was categorised as high (≥ 4 servings/day) or low (< 4 servings/day). This cut-off was based on a reference value from a previous study examining the association between UPF consumption and depression risk [22, 23]. Additionally, sex-specific quartiles were used in line with previous studies to further categorise UPF consumption.

### Cognitive function assessment

A series of cognitive tests was conducted to evaluate various cognitive processes in the ASPREE cohort. These tests included the Controlled Oral Word Association Test (COWAT) [24], which measures verbal fluency; the Symbol Digit Modalities Test (SDMT) [25], which assesses processing speed; the Hopkins Verbal Learning Test-Revised (HVLT-R) [26]), which evaluates verbal delayed recall; and 3MS [27], which measures a broad range of cognitive functions and was part of the eligibility assessment for ASPREE participants. Additionally, a composite measure was created to assess global cognitive function by summing the *Z* scores from the four cognitive tests (composite cognitive *Z* score). Cognitive assessments were measured at baseline, every other year during the trial phase, and annually thereafter. The primary outcome was the average of the cognitive function measurements (3MS, SDMT, COWAT, HVLT-R, and composite cognitive *Z* score), which were assessed from time zero (i.e. annual visit 3 of the ASPREE study) through to the fourth annual visit within the observational ASPREE- XT study ending in 2022 (up to a maximum of 11 ASPREE study annual visits).

### Assessment of potential confounders

We employed causal-directed acyclic graphs (DAGs) to pre-identify potential confounders (Appendix Figure 1). These included age, sex, ethnicity, smoking status, alcohol consumption, education, physical activity, living arrangements, social support, body mass index (BMI), multimorbidity (the presence of two or more chronic health conditions including hypertension, diabetes mellitus, dyslipidemia, chronic kidney disease, gastro-oesophageal reflux disease, pulmonary disease, gout, cancer, cardiovascular diseases and Parkinson’s disease), polypharmacy (the use of five or more prescription medications), metabolic syndrome (defined as the Adult Treatment Panel III (ATP III) diagnostic criteria as a composite measure of five variables (waist circumference, blood pressure, triglyceride, high-density lipoprotein cholesterol level, and blood glucose), depressive symptoms (CES-D 10 score), and composite cognitive *Z* score at time zero. Further details on the collection of demographic and clinical information can be found elsewhere [18].

### Statistical analysis

A summary description of participants’ characteristics, stratified by UPF consumption at time zero, is presented in Table 1. The marginal structural model was used to estimate the exposure effect [28]. The probability of being in the exposure group was predicted using logistic regression, with UPF consumption as the outcome variable, conditioned on prespecified covariates. Potential interaction terms were also examined. To create synthetic populations where the exposure group is independent of measured baseline confounders, we applied the Inverse Probability of Treatment Weighting (IPTW) approach. This method resulted in exposure and control groups with similar probabilities of receiving high or low levels of UPF, effectively simulating the characteristics of a target population [29, 30]. Stabilised weights were used to reduce the impact of selection bias and enhance the robustness of the estimations. To mitigate the influence of extreme weights, weight truncation (3%) was applied [29].

**Table 1:** Characteristics of participants at annual follow-up 3 (time zero)

Covariates	Control (UPF < 4 servings/day) (*n* = 7997)	Exposure (UPF ≥ 4 servings/day) (*n* = 3505)	SMD before IPW	SMD after IPW
Age at time zero	77.70 (4.00)	78.40 (4.30)	0.169	0.007
Education (> 12 years)	3341 (41.8)	1459 (41.6)	0.003	0.006
Race = White	7890 (98.7)	3478 (99.2)	0.056	0.002
BMI: mean (SD)	27.76 (4.6)	27.30 (4.36)	0.103	0.006
Smoking status: yes	212 (2.7)	50 (1.4)	0.087	0.003
Living status: living at home with family or friends	5258 (65.7)	2489 (71.0)	0.113	0.006
Alcohol use: yes	6080 (76.0)	2495 (71.2)	0.110	0.001
Sex = female	4675 (58.5)	1508 (43.0)	0.312	0.003
CES-D 10 score: mean (SD)	4.09 (3.92)	4.3 (3.96)	0.051	0.001
Multimorbidity: yes	6527 (81.6)	2889 (82.4)	0.021	0.007
Income: adequate	7396 (92.5)	3271 (93.3)	0.033	< 0.001
Metabolic syndrome: yes	4482 (56.0)	1820 (51.9)	0.083	0.001
Composite cognitive *Z* score at time zero: mean (SD)	0.12 (0.67)	0.02 (0.69)	0.148	0.006
Social support: good	7165 (89.6)	3131 (89.3)	0.009	0.002
Physical activity: moderate	4890 (61.1)	2149 (61.3)	0.003	0.006
Polypharmacy: yes	3358 (42.0)	1457 (41.6)	0.009	0.004

Note. Education: years of education (≤ 12 years and > 12 years); Smoking: current smoking status (yes vs no), Alcohol: current alcohol use (yes vs no), *CES-D 10*, Centre for Epidemiologic Studies-Depression 10 (CES-D 10) scale at time zero; Income: self-reported (adequate vs low or not adequate); Social support: defined based on the Lubben Social Network Scale (LSNS-6), and a value ≥ 12 out of 30 points was considered good social support) [32]; Multimorbidity: the coexistence of two or more chronic diseases, including hypertension, diabetes mellitus, dyslipidemia, chronic kidney disease, gastro-oesophageal reflux disease, pulmonary disease, gout, cancer, cardiovascular diseases, and Parkinson’s disease; Polypharmacy: the use of five or more prescription medications; Metabolic syndrome: based on the Adult Treatment Panel III (ATP III) diagnostic criteria as a composite measure of five variables (waist circumference, blood pressure, triglyceride, high-density lipoprotein cholesterol level, and blood glucose); Physical activity: usual intensity of physical activity in a typical week; low (never, rare or light) and moderate (moderate to vigorous); *SMD*, standardised mean difference; *IPW*, inverse probability weighting. The relationship between UPF exposure and cognitive scores at time zero showed significant negative associations between higher UPF consumption and cognitive scores (Appendix Table 2)

To estimate the association of UPF consumption on cognitive function, we fitted marginal structural models using generalised estimating equations (GEE). We assumed a Gaussian distribution with an identity link function and treated follow-up cognitive function scores as a continuous outcome variable, applying an autoregressive working correlation structure. We reported the estimated between-group mean differences with 95% confidence intervals and *p* values. Furthermore, we assessed the association between UPF consumption and cognitive decline among different subgroups, including sex, BMI, multimorbidity, metabolic syndrome, and aspirin intake.

Statistical significance was determined using a two-sided *p* value of less than 0.05. In sensitivity analyses, we used quartiles of UPF as exposure. Missing data in covariates were very low (< 5%) and addressed using the predictive mean matching method; no additional multiple imputation method was applied. Participants with missing diet data and those lacking cognitive function measures in the annual follow-up visits were excluded from the analyses.

An additional sensitivity analysis was conducted, excluding participants who had Parkinson’s disease at time zero to account for potential cognitive deficits associated with the condition. We also conducted a separate sensitivity analysis exploring the association between cognitive function and UPF consumption when categorised into sex-specific quartiles, consistent with previous literature. In addition, exploratory sex-disaggregated analysis was conducted, replicating the methods used in the main analysis. To demonstrate the extent to which the observed associations with ultra-processed food intake are independent of overall dietary quality, we also plotted UPF exposure against proxy measures of diet quality (the dietary inflammatory score [31] and total fruit and vegetable intake).

## Results

**Fig. 2:**
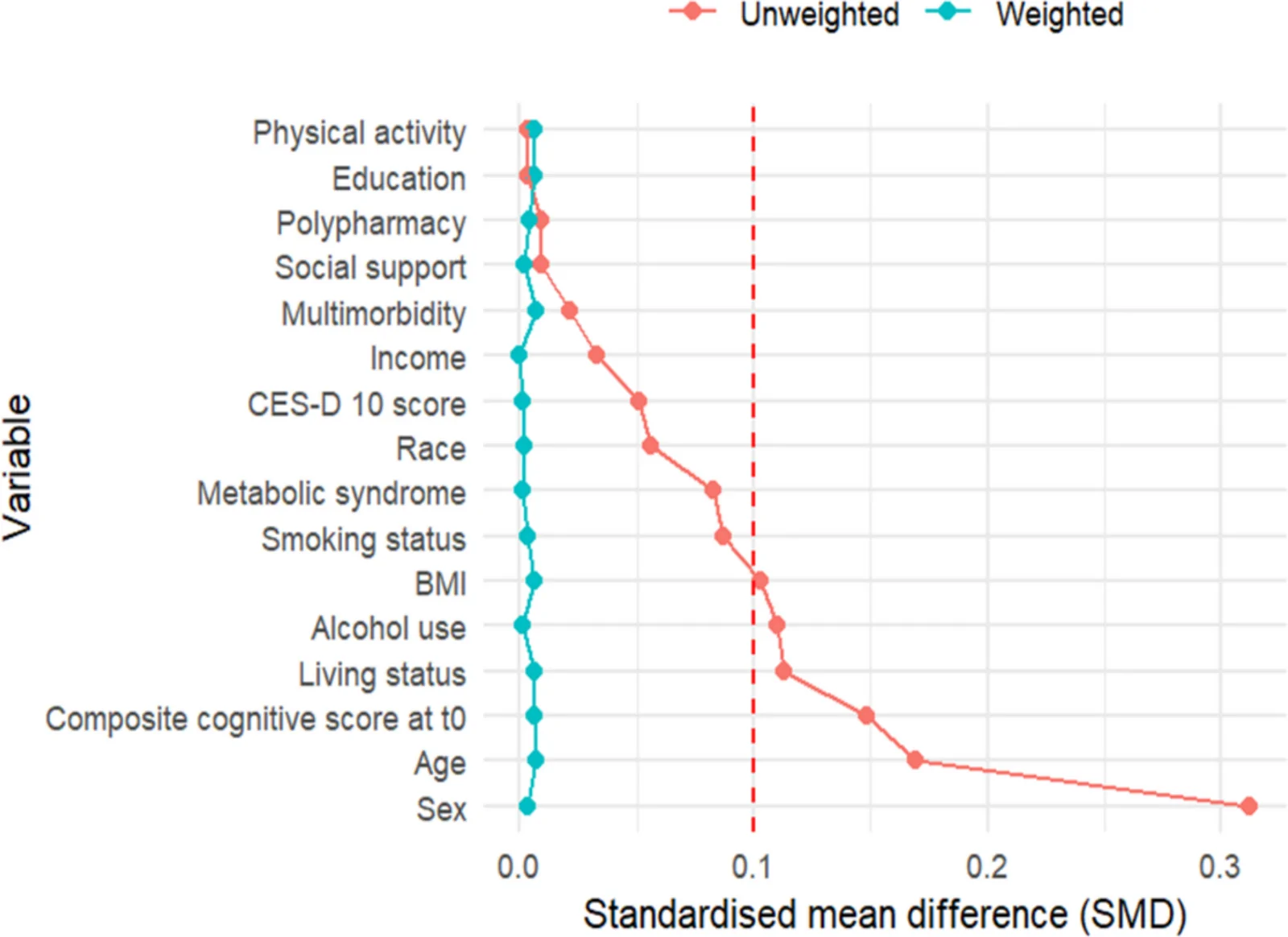
Absolute standardised mean difference (SMD) for covariates

Of the 12,578 participants who returned the annual visit 3 medical questionnaire in the ALSOP sub-study, 11,502 were eligible for this study. The mean (standard deviation) age of participants was 77.9 (4.1) years, and 6183 (53.8%) were female. Most participants had good social support (*n* = 8642, 89.9%), had two or more medical comorbidities (*n* = 7695, 81.8%), and drank alcohol (*n* = 8503, 74.6%). Almost half of the participants had polypharmacy use (4810, 42.0%) (Table 1). The mean UPF consumption was 3.44 ± 1.5 servings per day. UPF consumption was higher among males than females (3.73 ± 1.5 vs. 3.20 ± 1.4 servings per day). Of the total population, 3505 participants (30.5%) had high UPF consumption and were classified as the exposure group, and the remaining 7997 (69.5%) participants as controls. The median (interquartile range) follow-up was 5.6 ± 2.4 years. The baseline characteristics/covariates and potential confounders were well balanced after employing the IPTWs with a standardised mean difference of < 0.1 (Fig. 2 and Appendix Figure 2).

### Association of high UPF consumption and cognitive function

**Fig. 3:**
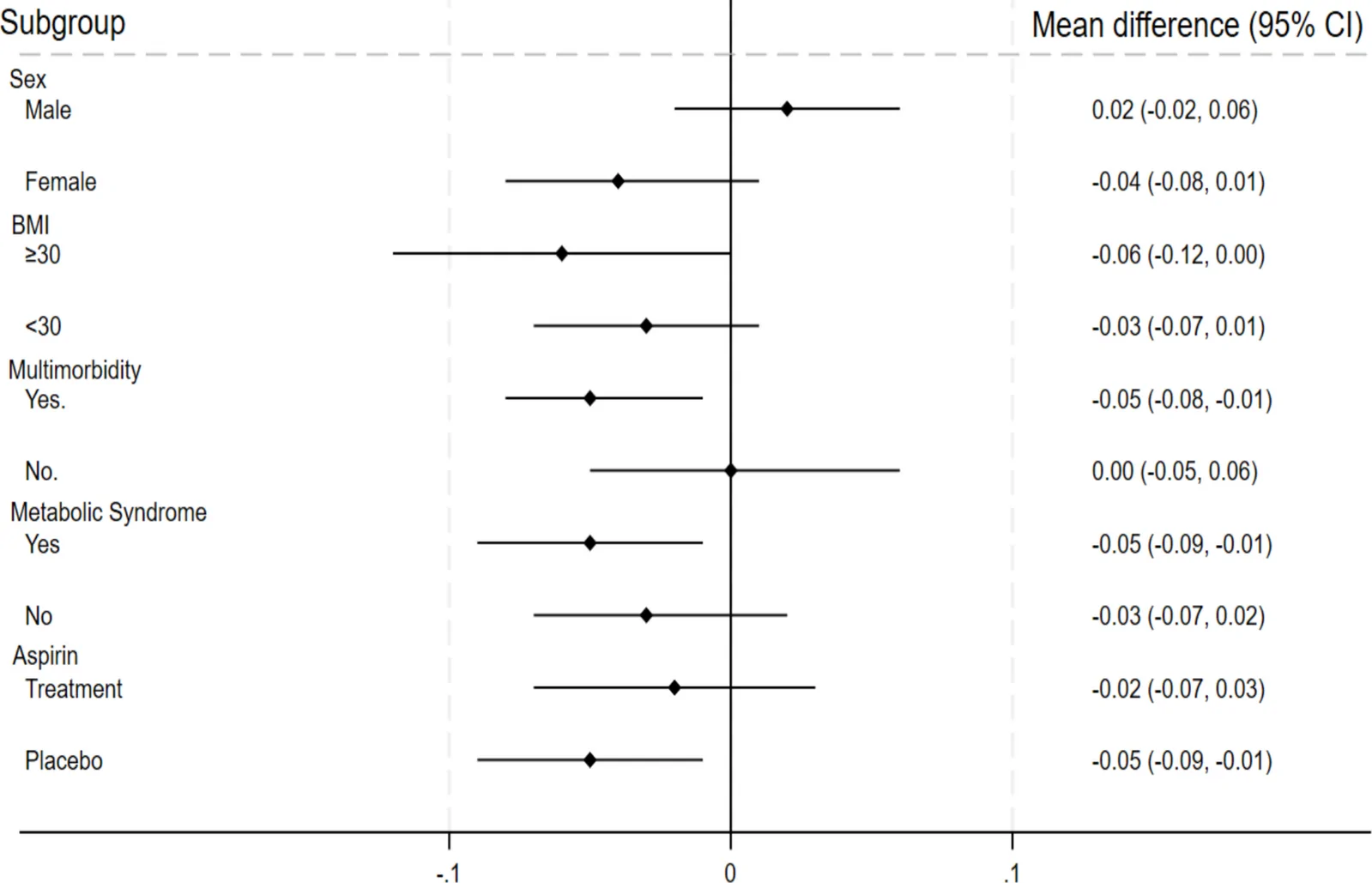
Mean difference of global cognitive function score by sub-groups

High UPF consumption was associated with lower 3MS (mean difference: −0.28, 95% CI (−0.52, −0.04)), COWAT (−0.23, 95% CI (−0.44, −0.03)), SDMT (−0.51, 95% CI (−0.94, −0.08)), and composite cognitive *Z* score (−0.04 (−0.07, −0.01)) (Table 2). However, it was not associated with lower HVLT-R (−0.12, 95% CI (−0.26, 0.02)). Subgroup analyses showed similar patterns, with significant negative associations between UPF consumption and cognitive function among those with metabolic syndrome and multimorbidity (Figure 3). Global cognitive scores over the follow-up period for high and low UPF are shown in Appendix Figure 3. Sensitivity analyses showed a significant reduction in the composite cognitive *Z* score in the fourth quartile of UPF intake consumption compared with the first quartile (−0.06 95% CI (−0.10, −0.02), *p* value = 0.007) (Appendix Table 4). Sensitivity analyses (excluding people with Parkinson’s disease and categorising the UPF exposure to quartiles) showed similar results (Appendix Tables 3 and 4). Effect modification of sex was assessed by testing a two-way interaction between UPF and sex (*p* < 0.001 for composite cognitive score). Although the sex by UPF interaction was significant, an exploratory sex-disaggregated analysis did not indicate sex-specific trends (i.e. no significant association between UPF and cognitive measures in male or female subgroups) (Appendix Table 5). In addition, UPF exposure was plotted against the dietary inflammatory score and total fruit and vegetable intake and used as a proxy for overall dietary quality (Appendix Figures 4 and 5).

**Table 2:** Mean difference in cognitive function scores between high and low UPF consumption (over a median follow-up period of 5.6 ± 2.4 years)

	Mean difference (95% CI)	*p* value	BH *p* value
Composite cognitive *Z* score	− 0.04 (− 0.07, − 0.01)	0.023	0.035
3MS	− 0.28 (− 0.52, − 0.04)	0.022	0.035
COWAT	− 0.23 (− 0.44, − 0.03)	0.028	0.035
HVLT-R	− 0.12 (− 0.26, 0.02)	0.096	0.096
SDMT	− 0.51 (− 0.94, − 0.08)	0.019	0.035

Covariates: age, sex, BMI, smoking, alcohol use, racial category, education status, living status, income, social support, multimorbidity, cognition score at time zero, CES-D 10 at time zero, metabolic syndrome, polypharmacy, and intensity of physical activity were included in the inverse probability weights. *BH*, Benjamini–Hochberg adjusted *p* value

## Discussion

This study prospectively examined the relationship between UPF exposure and cognitive function in older adults. Higher exposure to UPFs was associated with poorer cognitive function across several measures, including the 3MS, COWAT, SDMT, and the composite cognitive *Z* score, which assesses global cognition, verbal fluency, and processing speed [24–27]. In contrast, no association was observed between exposure to UPFs and the HVLT-R, which measures delayed recall and verbal learning.

These findings are consistent with recent prospective cohort studies from Brazil [16, 17]. Gomes Gonçalves et al. found that higher UPF use was associated with a 28% faster rate of global cognitive decline [17]. Similarly, Bhave et al. reported that a 10% increase in the relative intake of UPFs was associated with a higher risk of cognitive impairment in people free from cognitive impairment at baseline [16]. The present study extends this emerging evidence by examining additional cognitive outcomes, including verbal fluency and processing speed, and by situating these associations within an Australian context, where dietary patterns, the food system, and regulatory environment differ from those previously studied. The absence of the association between UPF exposure and HVLT-R may be due to differences in cognitive ageing and domain sensitivity to UPF exposure. HVLT-R is a measure of delayed recall and verbal learning, whereas the other cognitive measures reflect global cognition, executive function, and attention. Consistent with these results, previous studies have reported associations between exposure to UPF and measures of global cognitive function or executive function [15, 17].

### Potential mechanisms of action

Several potential mechanisms may explain the association observed in this study. UPFs are typically high in sodium, energy density, trans fats, and sugar, while being lower in fibre [13]. This nutrient profile has been linked to an increased risk of cognitive decline [33–35]. Moreover, UPFs are often deficient in bioactive compounds, such as polyphenols [36], which have demonstrated anti-inflammatory properties and have been associated with improved cognitive performance [37]. Beyond nutrients, UPFs contain additives (e.g. emulsifiers, artificial sweeteners, preservatives) that may induce inflammation, impair glucose tolerance, and disrupt the gut microbiome [13, 38–40].

Furthermore, UPFs are associated with cardiovascular and metabolic diseases [14, 41], which are known risk factors for cognitive impairment [42, 43]. Experimental evidence confirms that these foods can drive rapid metabolic disruption: in a controlled inpatient randomised crossover trial, adults allowed to eat ad libitum from an UPF menu consumed 500 kcal more per day and gained weight within 14 days compared with an unprocessed diet [44]. UPF exposure may also negatively influence cognition through pathways linked to depression. Specifically, high consumption of UPF has been related to increased risk of depression [22]. In turn, depression is associated with impairments in cognitive function [45]. Theoretically, the parallel risk pathways for the development and progression of neuropsychiatric disorders overlap with those driving the progression of medical disorders [46]. This is likely driven by neuroinflammatory processes, hypothalamic–pituitary–adrenal axis dysregulation, and changes in brain structure and connectivity [47].

### Strengths, limitations, and implications of this research

To our knowledge, this is the first study to investigate the relationship between UPF intake and cognition in a large, longitudinal Australian cohort. Further, this study had a relatively long follow-up and was a well-characterised sample that allowed for adjustment for many potential confounders. This study also has the following limitations. Although participants with dementia at baseline were excluded, it is possible that subtle, early cognitive changes influenced dietary behaviours at the time dietary data were collected. To mitigate the risk of reverse causality, we initiated the follow-up period in year 4, 1 year after the collection of dietary data. Despite that, the possibility of reverse causality remains an important concern, particularly given the long preclinical phase of cognitive decline [48]. Although there was some dropout during the follow-up period, we included participants with at least one composite cognitive score. The self-reported dietary data were collected using a questionnaire that was not validated and does not include serving sizes. Further, the dietary questionnaire was not designed to measure UPF. While we used an approach consistent with other studies [22], some misclassification remains possible. In addition, we classified supplemental drinks as UPFs. While these differ substantially from sugar-rich or artificially sweetened beverages, they meet the Nova criteria for UPFs.

This cohort study was derived from a primary prevention trial. The included sample may not represent the general population at this age. In the sensitivity analysis, the associations observed between UPF exposure and cognition were no longer significant in the sex-disaggregated analysis. This may be due to reduced power, as the overall effect sizes observed were modest in the main analysis. However, this could also be due to true sex differences in how UPF impacts cognitive ageing. Future research with larger stratified samples will be needed to determine whether the relationship between UPF consumption and cognitive decline truly differs by sex, or whether the attenuation observed here simply reflects a loss of statistical power. Future research is needed to establish the role of UPF consumption in middle-aged adults on the impact of later-life cognitive decline, as this is a key period for the development of risk factors for cognitive impairment later in life [11].

While the observed effect sizes were modest, they were comparable to available evidence [49]. Although the differences observed are unlikely to be clinically meaningful at the individual level, even small shifts in cognitive performance may have implications at the population level by increasing the proportion of older adults who transition into clinically relevant cognitive impairment over time. UPF exposure may represent one component of broader dietary and lifestyle patterns associated with cognitive ageing, rather than an isolated risk factor. In this context, the results support the growing evidence for multidomain approaches to cognitive decline [12, 50], including dietary guidance that promotes diets low in UPFs such as the Mediterranean, MIND, or DASH diets [11].

## Conclusion

This study contributes to a growing body of evidence linking UPF exposure to cognitive ageing. We found that in older Australian adults, higher exposure to UPFs was associated with poorer cognitive function on several cognitive tasks, including the 3MS, COWAT, SDMT, and composite cognitive *Z* score. At the public health level, this data suggests that shared approaches to prevention and promotion of medical and psychiatric disorders, focused on modifiable lifestyle factors, should be prioritised.

## Supplementary Information

Below is the link to the electronic supplementary material.

ESM 1 (DOCX 565 KB)

## Data Availability

Data cannot be shared publicly for legal and ethical reasons as data are part of a large ongoing observational cohort. Data are available from Monash University for researchers who meet the criteria at https://aspree.org/aus/for-researchers/.
