# Supplementary material for: The association between ultra-processed food exposure and cognition in older adults
Source: GeroScience. 2026 May 6;48(4):5149–60. doi: 10.1007/s11357-026-02269-w (PMC13575077; doi:10.1007/s11357-026-02269-w)
Supplement: Supplementary file 1 [file 11357_2026_2269_MOESM1_ESM.docx]

# **Appendix**

**Appendix. Table1** Classification of food and drink items according to the NOVA food classification system.

| **NOVA group** | **Food group** | **Food and drink items** |
| --- | --- | --- |
| Group 1 | Unprocessed or minimally processed foods, | Rice, pasta / noodles, fresh fruit, raw/salad vegetables, cooked green vegetables, cooked orange/ yellow / red vegetables (e.g. carrots, beets), potatoes, beans, and legumes (e.g. peas, lentils), yoghurt,  Red meat (not corned, pickled, or processed), Chicken, turkey or other poultry, eggs, Deep fried/battered fish, Salmon, mackerel or other oily fish (not including deep fried fish), White fish (not including deep fried fish),  Other seafood (e.g. prawns or shellfish), nuts, fruit juice, low-fat or skim milk, full-cream milk, water, coffee, tea, herbal tea |
| Group 2 | Processed culinary ingredients | Olive oil, butter, other vegetable oil, salt |
| Group 3 | Processed foods | Cheese, cream, cream cheese or similar, cooked, tinned or dried fruit, tinned fish (e.g. canned salmon, tuna, sardines). |
| Group 4 | Ultra-processed food | Soy or other non-dairy milk, malt drinks (e.g. Milo or Horlicks), hot chocolate, cordial, soft drink (e.g. regular Coke), diet Soft drink (e.g. diet Coke), Supplement drink (e.g. Ensure or Sustagen), sausages, processed meats (e.g. bacon, ham, corned beef or salami), potato chips or similar, sweet biscuits/ cakes, dark chocolate, milk chocolate, lollies or other sweets, hamburgers, pizza or 'fast' food, meat pies (sausage rolls etc), ice cream, frozen yoghurt or other dairy desserts, white bread, brown/multigrain bread, breakfast cereal/ oats, crackers / savoury biscuits. |

**Appendix. Figure 1.** The directed acyclic graph (DAG) for identifying the confounders

**Appendix. Figure 2:** Distribution plot for propensity score before and after weighting

**Appendix. Figure 3:** Global cognitive score over the follow-up data collection annual visits

**Appendix. Table 2:** Correlation matrix showing the relationship between UPF consumption and cognition scores at time zero

| Cognition score | UPF (serving/day) | P-value |
| --- | --- | --- |
| 3MS | -0.064 | <0.0001 |
| COWAT | -0.051 | <0.0001 |
| HVLT-R | -0.072 | <0.0001 |
| SDMT | -0.058 | <0.0001 |
| Composite cognitive Z score | -0.084 | <0.0001 |

**Appendix. Table 3:** Sensitivity analysis showing mean difference in cognitive function scores between high and low ultra-processed food consumption groups, excluding participants with Parkinson’s disease (n=167, over a median follow-up period of 5.6 ± 2.4 years).

|  | Mean difference (95% CI) | P value |
| --- | --- | --- |
| Composite cognitive Z score | -0.04 (-0.07, -0.01) | 0.018 |
| 3MS | -0.28 (-0.52, -0.04) | 0.023 |
| COWAT | -0.21 (-0.42, -0.00) | 0.049 |
| HVLT | -0.13 (-0.28, 0.01) | 0.060 |
| SDMT | -0.41 (-0.84, 0.02) | 0.064 |

**Appendix. Table 4:** Mean difference in global cognition score by sex-specific quartiles of ultra-processed food consumption (over a median follow-up period of 5.6 ± 2.4 years).

| Quartiles of UPF  consumption | Mean difference (95% CI) | P-value |
| --- | --- | --- |
| Quartile 1 | Reference |  |
| Quartile 2 | -0.04 (-0.08, -0.01) | 0.027 |
| Quartile 3 | -0.02 (-0.06, 0.02) | 0.379 |
| Quartile 4 | -0.06 (-0.10, -0.02) | 0.007 |

**Appendix. Table 5:** Sex-disaggregated analysis for mean difference in cognitive function scores between high vs low UPF consumption

|  | Male | | Female | |
| --- | --- | --- | --- | --- |
|  | Mean difference  (95% CI) | P-value | Mean difference  (95% CI) | P-value |
| Composite  cognitive Z score | 0.02 (-0.02, 0.06) | 0.369 | -0.04 (-0.08, 0.01) | 0.121 |
| 3MS | 0.1 (-0.31, 0.33) | 0.933 | -0.10 (-0.43, 0.23) | 0.544 |
| COWAT | -0.02 (-0.29, 0.26) | 0.907 | -0.14 (-0.46, 0.17) | 0.382 |
| HVLT-R | 0.18 (-0.01, 0.37) | 0.066 | -0.20 (-0.40, 0.00) | 0.054 |
| SDMT | -0.04 (-0.61, 0.54) | 0.902 | -0.53 (-1.15, 0.10) | 0.099 |

- The dietary inflammation score was calculated based on 18 components (17 food groups and one supplement group) according to the methods described by Byrd et al. <https://doi.org/10.1093/jn/nxz165>

**Appendix. Figure 4:** Scatter plot UPF by dietary inflammation score

**Appendix. Figure 5:** Scatter plot UPF by total fruit and vegetable intake
